# GhLCYε-3 characterized as a lycopene cyclase gene responding to drought stress in cotton

**DOI:** 10.1016/j.csbj.2023.12.024

**Published:** 2023-12-22

**Authors:** Kesong Ni, Xuke Lu, Shuyan Li, Fei Li, Yuexin Zhang, Ruifeng Cui, Yapeng Fan, Hui Huang, Xiugui Chen, Junjuan Wang, Shuai Wang, Lixue Guo, Lanjie Zhao, Yunxin He, Wuwei Ye

**Affiliations:** a Institute of Cotton Research of Chinese Academy of Agricultural Sciences / Research Base, Anyang Institute of Technology, National Key Laboratory of Cotton Bio-breeding and Integrated Utilization, Anyang 455000, Henan, China; b Hunan Institute of Cotton Science, Changde 415101, Hunan China

**Keywords:** Carotenoids, Cotton, Functional verification, Lutein, Reactive oxygen species (ROS), Lycopene cyclase

## Abstract

Drought stress significantly affects crop productivity. Carotenoids are essential photosynthetic pigment for plants, bacteria, and algae, with signaling and antioxidant functions. Lutein is a crucial branch product in the carotenoid synthesis pathway, which effectively improves the stress tolerance of higher plants. lycopene cyclase, a central enzyme for lutein synthesis, holds great significance in regulating lutein production. This research establishes a correlation between lutein content and stress resistance by measuring the drought resistance and lutein content of various cotton materials. To identify which crucial genes are associated with lutein, the lycopene cyclase family (LCYs) was analyzed. The research found that LCYs form a highly conserved family divided into two subfamilies, LCY-ε (lycopene ε-cyclase) and LCY-β (lycopene β-cyclase). Most members of the LCY family contain photoresponsive elements and abscisic acid elements. qRT-PCR demonstrates showed that most genes responded positively to drought stress, and *GhLCYε-3* was expressed significantly differently under drought stress. Virus-induced gene silencing (VIGS) assay showed that the content of *GhLCYε-3* was significantly increased with MDA and PRO, and the contents of chlorophyll and lutein were significantly decreased in pYL156 plants. The decrease in *GhLCYε-3* expression is speculated to lead to reduced lutein content in vivo, resulting in the accumulation of reactive oxygen species (ROS) and decreased drought tolerance. This research enriched the understanding of LCY gene family and lutein function, and provided a new reference for cotton planting in arid areas.

**Synopsis:**

Lycopene cyclase plays an important role in enhancing the ability of scavenging ROS and drought resistance of plants.

## Graphical Abstract

**Figure:**
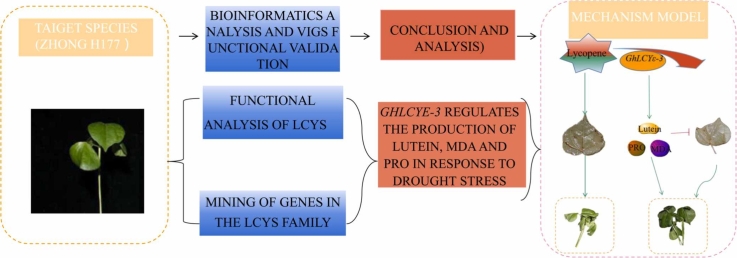

## 1 Introduction

In 1906, carotene, lutein and chlorophyll were successfully isolated from green leaves [1]. Carotenoids are isoprene metabolites derived from isoprene pyrophosphate (IPP) and its isomer, dimethylallyl diphosphate (DMAPP) [2]. The carotenoid synthesis pathway involves nine catalytic enzymes, including octahydro lycopene synthase (PSY), lycopene ε-cyclase (LCY-ε), lycopene β-cyclase (LCY-β), and 9-cis-epoxy carotenoid dioxygenase (NCED), etc. These fat-soluble pigments act as antioxidant stress protectors in plants [3]. For instance, when green plants are overexposed to sunlight, the photosynthetic system is damaged, and photosynthetic free radicals are quenched with carotenoids in the plant to protect the plant from strong light damage [4]. Carotenoids also play a crucial role in the drought resistance of higher plants. Studies have shown that radish (*Raphanus sativus* L) has a significant increase in the content of carotenoids and malondialdehyde (MDA) in the roots when subjected to drought stress [5]. Additionally, after treating the carrot taproot with PEG, it was found that the expression of *9-cis-epoxycarotenoid dioxygenase (DcNCED1)* and *DcNCED2* increased significantly under drought stress, and lutein levels exhibited varying degrees of increase. This indicated that NCED genes could respond to drought stress [6].

In plants, carotenoids are mainly divided into carotene and lutein, of which lutein accounts for 59% of the total carotenoids [7]. Lutein, a crucial branching product in the carotenoid synthesis pathway, is a yellow pigment closely related to the photosynthetic reaction center and plays a crucial role in plant removal of oxidative damage and response to drought stress [8]. lycopene cyclase, as a central enzyme for lutein synthesis, is of great significance for regulating lutein production. The lycopene cyclase family (LCYs) was initially identified in bacteria, exhibiting high conservation from bacterial to plant domains [9]. Subsequent experiments confirmed that the amino acid sequences of lycopene cyclase of plants and cyanobacteria are highly similar, and they are both composed of 400 amino acid residues and a polypeptide with a molecular weight of 43kD [10]. LCYs have been demonstrated in various plants, classified into two subfamilies, lycopene ε-cyclase and lycopene β-cyclase [11]. In 1996, the function of β and ε lycopene cyclases was analyzed, revealing that it has the function of regulating the proportion of downstream carotenoids produced [12]. The close relationship between lycopene cyclase and plant stress resistance has been demonstrated, with the overexpression of LCY-β enhancing tolerance to abiotic stresses in tomatoes [13].

Cotton, an important cash crop, is susceptible to various environmental stresses during its growth, and drought stress is one of the main abiotic stress factors affecting cotton yield. Drought stress not only affects the growth and development of cotton, but also severely weakens its productivity, and this impact is likely to expand further as global warming intensifies. Therefore, it is of great significance to the study cotton drought tolerance. Drought stress negatively affects plant morphological, physiological and biochemical processes [14]. Drought stress induces various responses in plants, including the regulation of osmotic substances to mitigate damage. At the cellular level, drought stress triggers Ca^2+^ signaling, reactive oxygen species (ROS), and abscisic acid (ABA) hormone-mediated signaling. Carotenoids act as signaling molecules, reducing oxidative damage and enabling plants to adapt to various stresses [15]. Carotenoids, as synthetic precursors of ABA, improve the drought resistance of cotton by regulating the production of ABA. Carotenoids are essential for survival in clearing reactive oxygen species ROS in the body [16]. Lutein has been observed to affect the functional chlorophyll antenna size of photosystem II, preventing ROS formation by inhibiting harmful substances by binding at site I of the plant's major LHCII complex [17]. Some carotenoid genes are involved in regulating plant responses to various biotic and abiotic stresses. Overexpression of the lycopene β-cyclase gene in tobacco enhanced the salt and drought tolerance of transgenic plants [18]. Despite extensive studies on lycopene cyclase in various species, its function in cotton has not been reported, making the study of lycopene cyclase in cotton necessary.

In this research, 12 drought-tolerant materials were selected to determine the content of lutein in cotton seedlings after the treatment with 15% polyethylene glycol (PEG 6000) and lycopene cyclase (LCYs) were mined in order to explore the relationship between lutein and drought resistance. The obtaied gene *GhLCYε-3* was subjected to a VIGS experiment, from LCYs, revealed increased wilting under drought stress, accumulated more ROS, faster water loss on leaves, increased malondialdehyde and proline, and decreased chlorophyll and lutein levels. The results provide a reference for the functional study of LCYs in other species and the breeding of drought-tolerant varieties of cotton.

## 2 Materials and methods

### 2.1 Experimental materials

12 materials were chosen to determination of the lutein content in cotton seedlings (Table 1), with 30 capsules per material and three replicates. PEG6000 (Solarbio,Beijing) served as the standard for simulating drought conditions [19]. The treatment concentration was set at 15% PEG6000 and the processing time was 3 days, based on our previous research results [15]. The 12 materials are drought-tolerant and are preserved by the Institute of Cotton Research of Chinese Academy of Agricultural Sciences. The seeds were placed vertically and evenly on cut filter paper in a tray, with the filter paper moistened either with ddH2O or a 15% PEG6000 solution. The filter paper is rolled into straight tubes, and the rolled filter paper (growth point down) is transferred vertically to a beaker containing 15% PEG6000 (adding PEG solution to 1/5 of the total beaker range) and ddH_2_O. The beaker is transferred to a biochemical incubator at 25 °C/24 h for dark germination, removed after 72 h of treatment, observed seed germination and recorded. Germination potential was calculated as the number of germinations divided by the total number, multiplied by 100% (germination was considered when the germ length reached half of the seed length).

**Table 1:** Determination of germination potential and lutein content of 12 materials.

Cotton materials	Germination potential (%)	Fresh weight (g)	Root length (cm)	Lutien Content（mg/g）
Chuang mian 22	84.4	0.2258	5.2724	0.0022
Guang zi 2	90.9	0.3067	6.1846	0.0022
Xin qiu 4	93.6	0.3280	7.8042	0.0061
Guang zi 1	90.9	0.3067	6.1846	0.2180
Zhong 9806	88.1	0.2611	4.6476	0.0240
G1286	76.9	0.3433	7.0857	0.0242
Han 242	96.7	0.3261	8.0409	0.0253
Xuan yu 9409	84.8	0.2925	7.6783	0.0341
Ji 228	88.2	0.3080	6.6417	0.0345
Lu 16	73.3	0.2363	4.7207	0.0358
High temperature resistance 2	90.1	0.2768	6.3167	0.0404
Zhong H177	93.0	0.3371	7.7571	0.0427

Uniform-sized cotton seedlings were preserved in liquid nitrogen. Due to the fat solubility of carotenoids, acetone extraction methods were used in this study [20]. The whole seedlings are placed in a pulverizer and the sample is ground thoroughly. Lutein was extracted from seedlings using 50% acetone, left to stand protected from light for 24 h and centrifuged for analysis. Absorbance photometric values at 470, 485, 642, 665 nm were measured using a microplate reader (BioTek Synergy HT, USA). Chlorophyll a (mgL^-1^)= 9.99A_665_-0.0872A_642_, Chlorophyll b(mgL^-1^)= 17.7A_642_-3.04A_665_, Lutein= (mgL^-1^) = 10.2A_470_-11.5A_485_-0.0036[a]− 0.652[b], Lutein content (mgL^-1^) = Lutein (mg·L^-1^) × Total volume of extract / Sample fresh weight (g).

### 2.2 Identification of LCYs gene family members

Genome sequences of four *Gossypium* species including *G. hirsutum*, ZJU; *G. barbadense*, HAU; *G. arboreum*, CRI; and *G. raimondii,* JGI were used to identify the gene family [21]. Protein sequences of tetraploid (*G. barbadense* and *G. hirsutum*) and diploid (*G. arboreum* and *G. raimondii*) cotton varieties were downloaded from Cotton FGD (https://cottonfgd.org) [22]. Additionally, genome datas of six other plants, *A. thaliana, Vitis vinifera (V. vinifera), Populus trichocarpa (P. trichocarpa), Theobroma cacao (T. cacao), Oryza sativa (O. sativa) and Zea mays (Z. mays)* were downloaded from the Phytozome V12.1 online site (https://phytozome-next.jgi.doe.gov/) [23]. The Hidden Markov models (HMMs) (version 3.0) profiles of (Pfam05834) were downloaded from online website Pfam (https://pfam.xfam.org/) to obtain the LCY proteins with default parameters, then we further screened the LCY proteins by using. In order to identify potential genes for the LCYs in the terrestrial cotton genome, genes with the conserved domain of the lycopene cyclase protein (PF05834) were screened using the hidden Markov model as a query file. The Pfam database was used for additional analysis (PF05834) to manually exclude genes with incomplete domains based on the conserved domain of the LCYs protein.

### 2.3 Phylogenetic evolutionary analysis of GhLCYs and prediction of cis-acting elements

The obtained LCYs sequences were submitted to CottonFGD (https://cottonfgd. net) to get physicochemical parameters like protein length, molecular weight (MWs), isoelectric points (pIs) and subcellular localization. Subsequently, 49 LCYs protein sequences were entered into MEGA7.0 ( https://www.megasoftware.net/) in order to examine the evolutionary relationships between LCYs. Phylogenetic trees for LCY proteins from four cotton species and six other species were constructed using the Neighbor Joining (NJ) method with default parameters in MEGA 7 software. The construction was based on the LG+G model, and 1000 bootstrap repeat sequences were utilized for robustness analysis [24]. The 2000 bp DNA sequence in the upstream region of the GhLCYs gene was obtained from the Cotton FGD website (https://www.cottonfgd.org/). The Plant CARE website (http://bioinformatics.psb.ugent.be/webtools/plantcare/html/) was used to predict the *cis-acting* elements in the promoter region of LCYs genes. The *cis-acting* elements schematic was constructed by TBtools software.

### 2.4 Four cotton motif composition analysis and chromosome location

The protein sequences were entered into the online MEME software to predicted the LCYs conserved motif sequence, The analysis was set to allow a maximum of 15 motifs, and other parameters were kept at default settings (http://meme-suite.org/tools/meme) [22]. Genome-wide annotation files and General Feature Format (GFF) files for four different cotton cultivars were acquired from CottonFGD (http://www.cottonfgd.net/). The TBtools (TBtools-ll Toolbox for Bioologists v1.108, parameter default https://github.com/CJ-Chen/TBtools/releases) was used by default to locate and analyze the LCYs gene chromosomes of fou r cottons [25]. FPKMs of LCYs are collected from Gossypium Resource and Network Database online database (https://grand.cricaas.com.cn/page/tools/expressionVisualization). The expression of diferent GhLCYs and GbLCYsin cotton response to salt (400 mM NaCl), drought (20% PEG), cold (4 ℃) and heat (37 ℃) for diferent time points (1, 3, 6 and 12 h) were analyzed.

### 2.5 LCYs protein interaction prediction

GhLCYε-3 protein sequences were downloaded from CottonFGD (https://cottonfgd.net). GhLCYε-3 protein sequences were entered into online websites (https://cn.string-db.org/) where prediction protein interactions, and finally the most similar basic sequences were selected for plotting [26]. I-TASSER was used to identify 8 proteins of GhLCYs, enter protein sequences into online website prediction (https://zhanglab.ccmb.med.umich.edu/I-TASSER/), and selected the most similar to plot [27]. 8 GhLCYs protein sequences were entered into the SOPMA (https://npsa.lyon.inserm.fr/cgi-bin/npsa_automat.pl?page=/NPSA/npsa_sopma.html) online website to predict protein structures, with default parameters.

### 2.6 Plant treatment and fluorescence quantitative detection of GhLCYs expression analysis

Genome-wide annotation files and General Feature Format (GFF) files for four different cotton cultivars were acquired from CottonFGD [28]. The seeds were sown in sand and incubated at 26 °C for 16 h during the day and 8 h at night. To investigate the effect of PEG on GhLCYs expression, 15% PEG6000 treatment was used. 0% PEG6000 treatment was used as a control and samples were taken separately until the phenotype of the treatment appeared. True leaf samples were frozen at − 80 °C, and RNA extraction was carried out using the Adlai EASYspin Plus kit, employing the lysate milling method. As a template for qRT-PCR, RNA was extracted and reverse-transcribed into cDNA. To determine the response of genes to drought, 8 genes were selected under pressure. GhLCYs qRT-PCR primer sequences were created by Premier V6 (http://www.premierbiosoft.com/) design (Supplementary Table S1). The PCR production size range for primers is set to 50–100, and all other parameters are default. Three biological replicates were used in qRT-PCR experiments on the Bio-Rad 7500 Fast Photo-PCR platform in accordance with the manufacturer's instructions for TransStart Top Green qPCR Supermix (TransGene Biotech Co., LTD, Beijing, China). The reaction conditions were predenatured 95 °C, 300 s, denatured 95 °C for 20 s, annealed 55 °C for 20 s, extension 72 ℃ for 20 s, and the total cycle was set to 40 times. The 2-^ΔΔCt^ standardized method was used to determine the relative expression level of genes [29]. SPSS software (IBM SPSS Statistics 21, https://www.ibm.com/spss) is used for statistical analysis of data confidence testing. *GhUBQ7* served as the internal control and was stably expressed in cotton plants, remaining unaffected by treatment or genotype [30].

### 2.7 Preliminary functional verification of GhLCYs

Previous studies have shown that VIGS technology is widely used in cotton [31]. To explore the function of Lycopene ε cyclase (*GhLCYε-3*), a 300 bp silent fragment (Supplementary Table S1) was designed (https://vigs.solgenomics.net/) and Ten genes downstream of *GhLCYε-3* were fluorescently assayed for changes, and its primers were added in (Supplementary Table 1). The constructed VIGS vector was transformed into Escherichia coli (DH5-α competent cells, weidi), and the plasmid was extracted after the bacterial solution was sequenced correctly. The pYL156 vector was maintained by the laboratory for a long time. The silenced fragments were attached to the pYL156 vector preserved in this experiment, and three treatments were designed with a negative control (CK), a positive control (pYL156), and a silencing plant pYL156-*GhLCYε-3*. The bacterial fluid was injected into the leaves of Zhong H177, followed by incubation in a dark chamber at 25 °C for 24 h, transitioning to 25 °C with a 16 h light/8 h darkness cycle. Both VIGS plants and pYL156 plants were treated using PEG (PEG treatment for 3 h), and samples were collected upon leaf wilting and stored at − 80 °C.

### 2.8 Proline (Pro) and malondialdehyde (MDA) content detection

The leaf to be measured is placed on the instrument, and the chlorophyll II content of the leaf is determined using a content analyzer (Konica Minolta China Investment Co., Ltd.). The leaves of the pYL156 plant and the pYL156-*GhLCYε-3* plant were used to determine chlorophyll with three biological replicates per sample. The determine of chlorophyll II content is performed by a content analyzer (Konica Minolta (China) Investment Co., Ltd.). Leaves of pYL156 and pYL-*156-GhLCYε-3* plants were used to determine PRO and MDA. Take 0.1 g of the sample to be tested, grind the leaves separately, and then determine the content of Pro and MDA [32] with proline (Pro) and malondialdehyde (MDA) detection kits (Nanjing Institute of Bioengineering) according to the instructions [32]. Hydrogen peroxide (H_2_O_2_) was detected through staining with diaminobenzidine (DAB) after reaction, and ROS accumulation under drought stress was measured in leaves of plants pYL156 and pYL156-GhLCYε-3 (DAB) [33]. The leaves are placed in 1 g/L DAB staining solution, allowed to stand for 12 h, and decolorized with absolute ethanol [34]. The staining of the leaves was observed and recorded, and the black particles on the leaves were ROS [34].

### 2.9 Statistical analysis

A minimum of three biological replicates was considered necessary for each dataset. The data was analyzed using a single factor completely randomized trial, with the least significant difference (LSD) test employed to determine significance, denoted as *P < 0.05 or * *P < 0.01.

## 3 Results and analysis

### 3.1 Germination test and PEG tolerance evaluation of 12 cotton germplasm

**Fig. 1:**
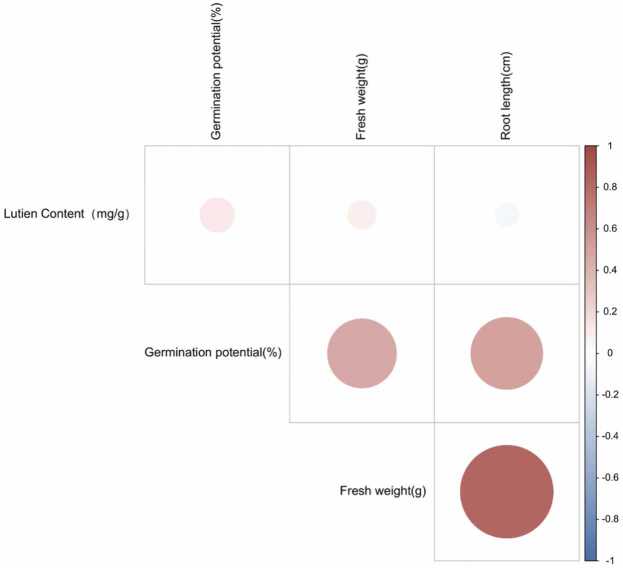
Correlation between germination, root length, fresh weight and lutein content of 12 cotton materials. Red shading indicate a positive correlation. Blue shading represents the strength of the negative correlation coefficient, with a closer absolute value to 1 indicating a stronger correlation.

Existing studies have shown that lutein is closely related to stress resistance. As shown in Table 1, the content of Zhong H177 lutein in 12 materials was high, and the germination potential, fresh weight root length were also at a high level. Therefore, Zhong H177 was selected for the next step of research. Fig. 1 illustrates that, during normal cotton germination, there exists a positive correlation between germination potential, fresh weight, and lutein, while root length shows a negative correlation with lutein (Fig. 1). To pinpoint the key gene responsible for regulating lutein, an analysis was conducted on the lycopene cyclase family, which governs lutein synthesis.

### 3.2 Identification and characterization of lycopene cyclase genes in four cotton species

The target genes were put into the CottonFGD (https://cottonfgd.net//) website, and the domain screen finally obtained 24 LCYs genes, 8 *Gossypium hirsutum* (GhLCYs), 8 *Gossypium barbadense* (GbLCYs), 4 *Gossypium arboreum* (GaLCYs), and 4 *Gossypium Raimondii* (GrLCYs), LCYs are highly conserved in the process of evolution. Screening in Arabidopsis thaliana using the domain, it was found that there were 3 genes LCYs in Arabidopsis, *AT2G32640 (ATLCYβ-1), AT3G10230 (LCYβ-2), AT5G57030 (ATLCYε-1)*, after comparing the Arabidopsis sequence with LCYs in cotton, the 24 LCYs identified in four cotton species were divided into two groups of LCY-β and LCY-ε. The LCY-β group exhibited a length ranged from of 487 to 497 amino acids (Table 2), while the length of LCY-ε group ranged from 497 to 769. Based on the gene's chromosomal position, we renamed it in the four cotton genomes. The isoelectric point, protein molecular weight, protein length and subcellular location of these genes were predicted and analyzed. The isoelectric points of the proteins encoded by LCYs ranged from 5.732–7.492 in the LCY-ε group, 7.864–8.526 in the LCY-β group, 55.469kD∼86.48kD in the LCY-ε group, and 54.639kD∼56.441kD in the LCY-β group. The protein sequences encoded by LCYs ranged from 497kD to 769kD amino acids. Subcellular localization prediction showed that LCY-ε was distributed in chloroplasts, and LCY-β group were distributed in chloroplasts and chromosomes.

**Table 2:** Analysis of physical properties of LCYs genes in four cotton species.

Gene ID	New ID	Protein length （aa）	Protein molecular weight（kDa）	Isoelectric point	protein hydrophobicity	Subcellular localization
*GB_A05G0316.1*	*GbLCYε-1*	535	60.124	6.204	-0.016	chloroplastic
*GB_D05G0318.1*	*GbLCYε-3*	535	59.888	5.937	-0.016	chloroplastic
*GB_D07G0130.1*	*GbLCYε-4*	769	86.480	6.920	-0.379	chloroplastic
*GB_A07G0122.1*	*GbLCYε-2*	497	55.469	7.490	-0.141	chloroplastic
*GH_A05G0311.1*	*GhLCYε-1*	535	60.125	5.986	-0.016	chloroplastic
*GH_D05G0316.1*	*GhLCYε-3*	535	59.904	5.732	-0.011	chloroplastic
*GH_A07G0130.1*	*GhLCYε-2*	553	61.683	7.492	0.066	chloroplastic
*GH_D07G0137.1*	*GhLCYε-4*	533	59.837	7.152	-0.037	chloroplastic
*Ga07G0136.1*	*GaLCYε-2*	532	59.704	6.722	-0.074	chloroplastic
*Ga05G0329.1*	*GaLCYε-1*	535	60.138	6.204	-0.016	chloroplastic
*Gorai.001G012900.1*	*GrLCYε-1*	532	59.662	6.637	-0.030	chloroplastic
*Gorai.009G032900.1*	*GrLCYε-2*	540	60.428	6.139	-0.012	chloroplastic
*GB_D09G1109.1*	*GbLCYβ-3*	497	56.441	7.892	-0.130	chloroplastic
*GB_A09G1262.1*	*GbLCYβ-1*	497	56.189	8.006	-0.127	chloroplastic
*Gorai.006G113300.1*	*GrLCYβ-1*	497	56.159	8.134	-0.106	chromoplastic
*Ga09G1139.1*	*GaLCYβ-1*	497	56.246	8.135	-0.128	chromoplastic
*GH_D09G1099.1*	*GhLCYβ-3*	497	56.235	7.864	-0.134	chromoplastic
*GH_A09G1145.1*	*GhLCYβ-1*	497	56.203	8.006	-0.126	chromoplastic
*GH_D13G0022.1*	*GhLCYβ-4*	497	55.777	7.946	-0.136	chloroplastic/chromoplastic
*GH_A13G0026.1*	*GhLCYβ-2*	497	55.873	7.938	-0.123	chloroplastic/chromoplastic
*Ga13G0026.1*	*GaLCYβ-2*	487	54.639	8.526	-0.102	chloroplastic/chromoplastic
*Gorai.013G002400.1*	*GrLCYβ-2*	497	55.817	7.938	-0.131	chloroplastic/chromoplastic
*GB_D13G0043.1*	*GbLCYβ-4*	497	55.803	7.938	-0.132	chloroplastic/chromoplastic
*GB_A13G0022.1*	*GbLCYβ-2*	497	55.845	7.938	-0.122	chloroplastic/chromoplastic

### 3.3 Conservative motif analysis of LCYs proteins

**Fig. 2:**
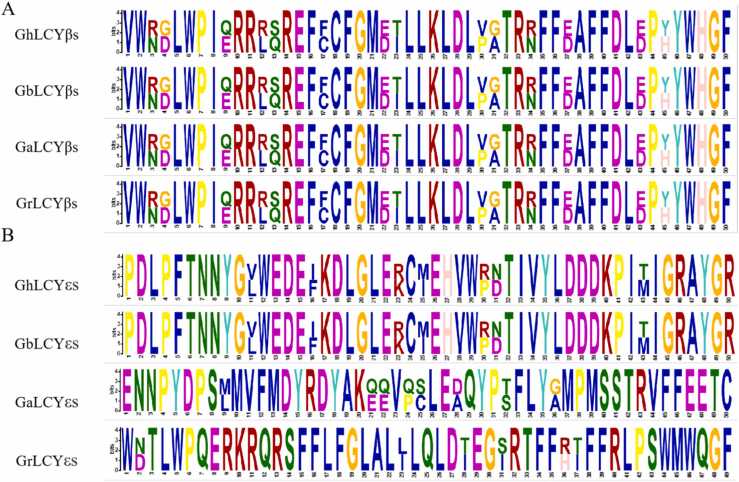
Conservative motifs analysis of LCYs sequences. Annotation：(A) and (B) representconserved basal sequences of four cotton species in the LCY-β group and LCY-ε group, respectively.

Conserved motifs are essential for gene function studies [35]. Four important presumed conserved motifs were identified for LCY-β and LCY-ε based on the conserved motifs in the four cotton species. The conservative motifs of the LCY-β and LCY-ε groups in the four cotton varieties were delineated. Despite these differences, numerous highly similar motifs were identified among LCYs in the four cotton species (Fig. 2). The LCY-ε group exhibited 36–50 similar conserved motifs, while the LCY-β group displayed 50 similar conserved motifs, suggesting a higher level of conservation in the LCY-β group in these species. It was clearly shown that LCYs in *Gossypium barbadense* and *Gossypium hirsutum* had higher basic similarity, possibly due to more complete gene copies of cotton in tetraploid. Our results showed that LCYs in four cotton species were highly conserved.

### 3.4 Phylogenetic analysis of LCYs

**Fig. 3:**
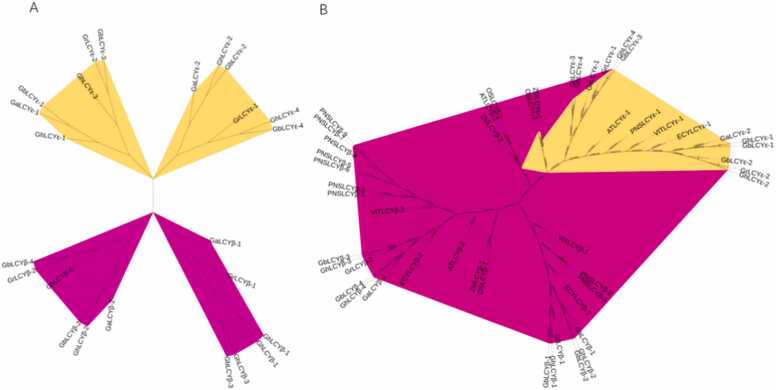
A: Evolutionary analysis of LCY family members in four cotton species; B: Phylogenetic relationships of 49 LCYs proteins form *G. hirsutum, G. barbadense, G. arboreum, and G. raimondii, A. thaliana, O. sativa, V. vinifera, P. trichocarpa, T. cacao, O. sativa, and Z. mays*.

Four intra-cotton phylogenetic trees were constructed to study LCY kinship (Fig. 3). Total of 49 protein sequences were derived from *G. hirsutum, G. barbadense, G. arboreum, and G. raimondii, A. thaliana, O. sativa, V. vinifera, P. trichocarpa, T. cacao, O. sativa, and Z. mays* were used to construct the interspecific tree. The phylogenetic tree is divided into LCY-ε and LCY-β groups, each with 12 genes in four cotton species, which is also divided into two groups, with 31 genes in group 1 and 18 genes in group 2 in 10 species. Among them, 8 LCYs genes were identified in *Gossypium barbadense,* 8 in *Gossypium hirsutum,* 4 in *Gossypium raimondii,* 4 in *Gossypium arboreum,* 3 in *Arabidopsis,* 3 in *Oryza sativa,* 10 in *Populus trichocarpa,* 3 in *grapes,* 3 in *Theobroma cacao and 3 in Vitis vinifera*. Notably, the phylogenetic trees depicted a distinct separation between monocots and dicots, emphasizing the highly conserved characteristics of LCYs across the 10 species.

### 3.5 Chromosome location of LCYs

**Fig. 4:**
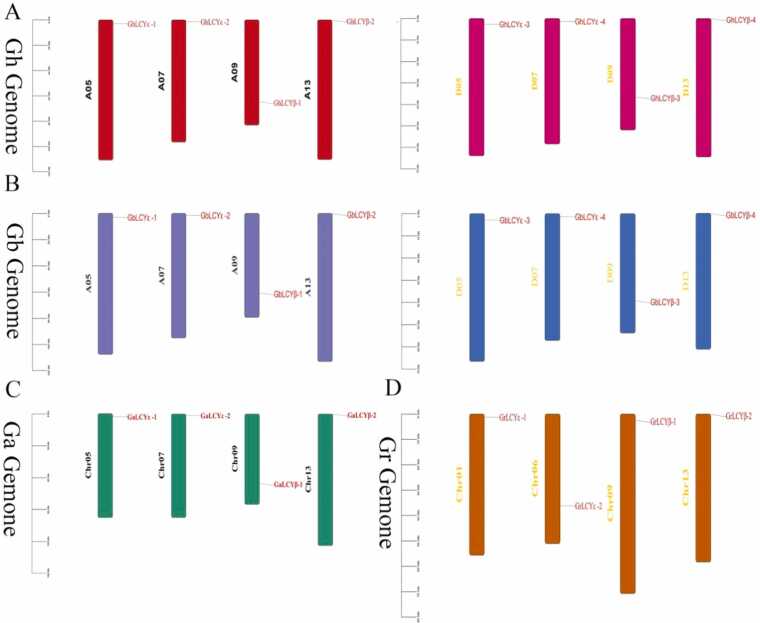
Chromosome distribution of LCYs gene family in four cotton species. Annotation (A) (B) (C) (D) Chromosomal location of LCYs on chromosomes in *G. hirsutum、 G. arboreum*、 *G. barbadense*.and *G. raimondii*.

The chromosomal arrangement of genes plays a pivotal role in the development and evolution of an organism's features [36]. The LCY-β group was distributed on chromosomes 09 and 13, and the LCY-ε group was distributed on chromosomes 05 and 07 except for *Gossypium raimondii*. In both *Gossypium barbadense* and *Gossypium hirsutum*, 4 genes were evenly distributed in subgroup A and subgroup D, and demonstrating a uniform arrangement. Similarly, in *Gossypium arboreum* and *Gossypium raimondii*, exhibited an even distribution of 4 genes across four chromosomes. In particular, except for *Gossypium raimondii*, the genes of the other three cotton varieties are distributed on chromosomes 05 and 07. As shown in Fig. 4 the distribution of genes in the cotton species, *Gossypium hirsutum*, *Gossypium arboreum*, *Gossypium arboreum* are similar in chromosomal location, suggesting that LCYs were conserved during evolution. However, the LCYs gene of Gossypium raimondii displayed a different pattern, with distribution across chromosomes 01, 06, 09, and 13. Notably, the gene distribution on chromosomes 06 and 09 in Gossypium raimondii differed from that in other cotton species, indicating a phenomenon of chromosome reversal.

### 3.6 Structural analysis of motif and cis-acting elements of LCYs

**Fig. 5:**
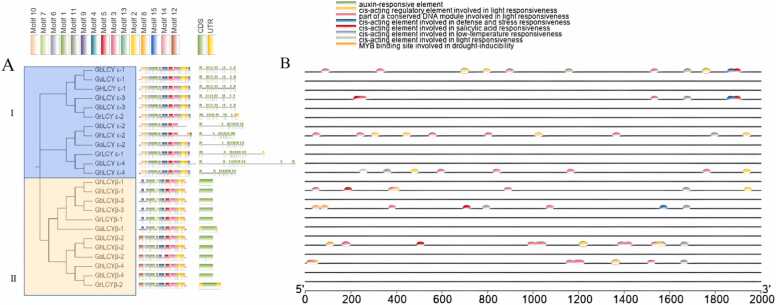
Conservative motifs **and*****cis-acting*****elements** of LCY gene family from *G. hirsutum, G. barbadense*, *G. raimondii*, and *G. arboreum.*

Exon, intron and encoded protein distribution patterns are reportedly employed for evolutionary study at various taxonomic levels [37]. In our investigation of the potential structural development of LCYs, we constructed a phylogenetic matrix and conducted a motif association analysis (Fig. 5). The results reveal that class Ⅰ (LCY-ε) contains 9–13motifs and all have the same motif except *GbLCY ε-2.* Meanwhile, the class Ⅱ (LCY-β) contains 11–12 motifs, but they have up to ten similar motifs. The LCY-ε and LCY-β groups were found to have 6–10 similar motifs, indicating the conservation of LCYs during evolution. Similar exon and intron patterns were identified in the LCY-ε group. Interestingly, the LCY-β group displayed almost no introns, suggesting its ability to respond more swiftly to stress. To further understand the response of LCYs genes to stress, PlantCARE was used to predict the LCYs promoter region. It was observed that most of the genes in LCYs contain photoreactivity, salicylic acid reactive MYB, drought, low temperature, some elements of defense stress response. Fig. 5 illustrated that the LCYε group contains more photosensing elements, accounting for about 8.0% of the total element, while the LCY-β group has more elements involved in defense and stress response, accounting for 7.6% of the total element.

### 3.7 Analysis of expression patterns of LCYs

**Fig. 6:**
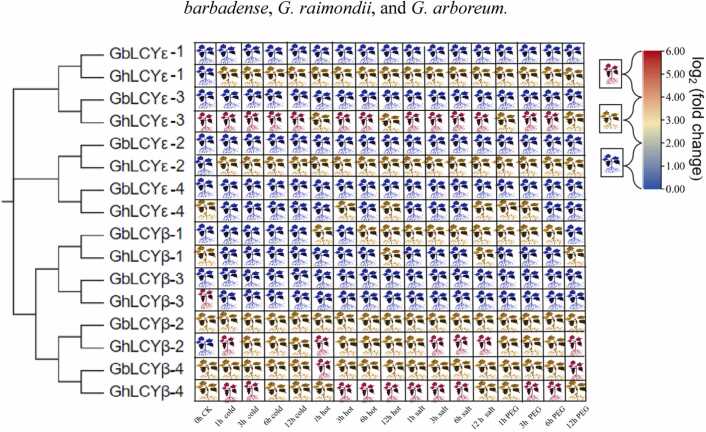
Analysis of expression patterns for LCYs genes in response to abiotic stresses. Annotation：.

To investigate the response mechanism of LCYs to abiotic stressors, FPKM (Replace with log_2_) of LCYs were gathered from an online database. The Gossypium Resource and Network Database were used to assess the expression profiles of these genes under various stresses (cold, heat, salt, and PEG), build a phylogenetic matrix, and correlate the expression heat map (Fig. 6). The findings indicated that multiple LCYs genes corresponded to various abiotic stresses, including *GhLCYε-1, GhLCYε-2, GhLCYε-3, GbLCYβ-2, GhLCYβ-2, GhLCYβ-4 and GbLCYβ-4*. In particular, the expression level of *GhLCYε-3* gene showed significant differences.

Color indicates Gene expression levels, red indicates high gene load, significantly differential expression. Yellow represents medium expression, blue expression is low expression or no expression.

### 3.8 Interaction network of LCYs proteins

**Fig. 7:**
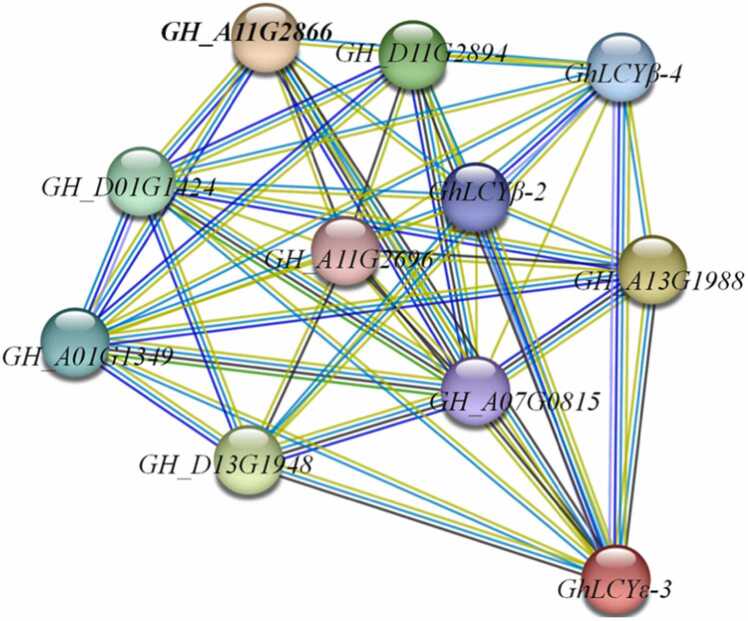
Protein–protein interaction analysis of LCYs proteins. Annotation: 10 protein in terrestrial cotton interact with the GhLCYε-3.

Protein-protein interaction analysis can reveal unknown functions of proteins [38]. Based on cotton protein sequence search, the interaction network of LCYs in cotton was analyzed (Fig. 7). 11 protein (Contains GhLCYε-3) interactions were identified, such as: Zeta-carotene desaturase (GH_A11G2866, GH_A13G1988, GH_D13G1948, GH_D11G2894)、Prolycopene isomerase (GH_D01G1424,GH_A01G1349), GhLCYβ-4 (GH_D13G0022), GhLCYβ-2 (GH_A13G0026), Phytoene synthase (GH_A07G0815) and Violaxanthin de-epoxidase (GH_A11G2696). This suggests that GhLCYε-3 may regulate the synthesis of lutein and ABA by interacting with GhLCYβ-2 and GhLCYβ-4 to complete the response to drought stress.

### 3.9 Three-dimensional (3D) structure prediction of GhLCYs proteins

**Fig. 8:**
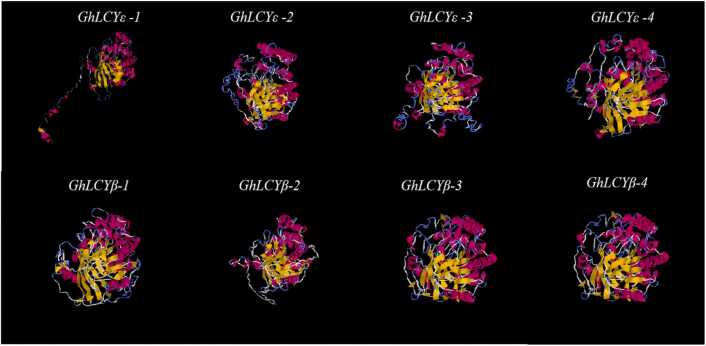
3D structure of 8 GhLCYs proteins. α-helices are indicated by red, β-strands are indicated by yellow, and random coils are indicated by blue. Annotation:α-helices are indicated by red, β-strands are indicated by yellow, and random coils are indicated by blue.

I-TASSER was used to obtain the 8 GhLCYs proteins structures (Fig. 8). Interestingly, the structure of GhLCYs proteins is mostly similar, while GhLCYε-1 has a special protein conformation. The structures of GhLCYs proteins consist of α-helices, β-strands and random coils. Specifically, the LCY-ε group exhibits α-helices ranging from 40.69% to 42.21%, random coils from 36.02% to 38.88%, and β-strands from 15.51% to 17.64%. On the other hand, the LCY-β group features α-helices ranging from 38.23% to 40.64%, random coils from 37.22% to 42.25%, and β-strands from 15.90% to 17.30%.

### 3.10 Expression patterns of s under drought stress

**Fig. 9:**
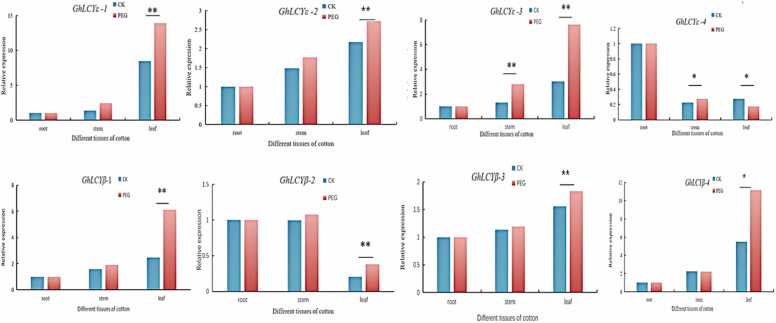
Analysis of GhLCY gene family expression under two treatments (CK and PEG) Annotation:* *P ≤ 0.01,*P ≤ 0.05.

The fluorescence quantitative results showed that the gene expression of GhLCYs was higher in leaves, and under normal growth conditions, such as *GhLCYβ-3* and *GhLCYβ-4* expression tended to stabilize in stems. Under PEG stress, there were significant differences in expression such as *GhLCYε-1、 GhLCYε-3、GhLCYβ-1* and *GhLCYβ-4*. Fig. 9 illustrates that *GhLCYε-3* and *GhLCYε-1* are expressed much higher after treatment than before treatment. In particular, the expression of *GhLCYε-3* gene increased by about 2 times compared with before treatment, and the expression level in the stem was significantly increased, so we selected *GhLCYε-3* gene for further study.

### 3.11 Cotton plants with the GhLCYε-3 gene silenced by VIGS were sensitive to PEG stress

**Fig. 10:**
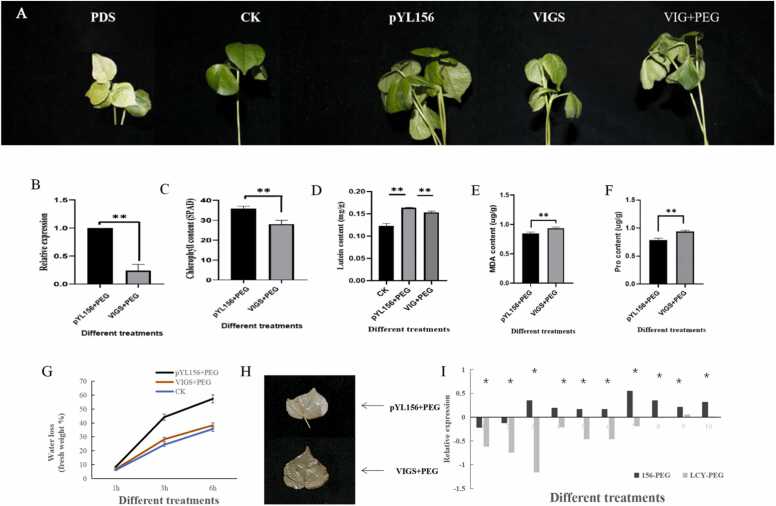
(A) Phenotype of cotton after silence of *GhLCYε-3* gene under stress. PDS: positive control; CK: control plant; pYL156 plants: negative control; VIGS: pYL156 *GhLCY ε- 3* plants; VIGS+ PEG: pYL156 *GhLCY ε- 3* plants+ PEG (B) Silencing efficiency of *GhLCYε-3* (C) Chlorophyll content of pYL156 +PEG and VIGS + PEG leaves under drought stress (D) Lutein content of pYL156 +PEG and VIGS + PEG leaves under drought stress (E) (F) MDA and Pro content in pYL156 + PEG and VIGS + PEG leaves. (G) Water loss rates of CK, pYL156 +PEG and VIGS+PEG cotton leaves under drought (H) DAB staining (I) Expression of 10 genes downstream of lutein. Annotation:* 0.01 <p < 0.05, P * * < 0.01.

VIGS was used to verify the effect of *GhLCYε-3* under drought stress. In Fig. 10 A VIGS plants withered more severely than control group (CK). Fig. 10 B shows a 63% decrease in the VIGS plant pYL156:*GhLCYε-3*. After silencing *GhLCYε-3*, we found that chlorophyll and lutein content were significantly reduced in silent plants. A decrease of about 27.2% in chlorophyll and 6.7% in lutein was observed, a ratio of about 4 multiples (Fig. 10 C and 10D). Biochemical indexes MDA activity and Pro activity were measured for CK and VIGS plants, revealing a significant increase in the content of MDA and Pro in VIGS plants. Additionally, the speed of leaf water loss was observed to be faster in VIGS plants (Fig. 10 E and 10 F). Furthermore, the expression of lutein-related genes downstream of VIGS plants is down-regulated, such as:PP2C8, AIP1, CYP97C1, CYP97A3, ABF2, PP2C, AIP1, ABF2, BHY, ABI5in (Fig. 10 I). Fig. 10 I showed that the reactive oxygen species of *GhLCYε-3* VIGS plants increased. The results showed that the reactive oxygen species scavenging system of *GhLCYε-3* VIGS plants was weakened, and the drought tolerance of cotton was reduced.

## 4 Discussion

Abiotic stress significantly impacts on the productivity of cotton. Lutein, known for its ability to remove ROS, plays a crucial role in plants' response to abiotic stress. The identification of LCYs, key genes in the biosynthetic pathway of lutein, is of great significance for improving the stress tolerance of crops. In order to explore the relationship between lutein and stress resistance, LCYs that control lutein synthesis were selected for further research. The research analyzed the expression of LCYs in four cotton species with conserved motifs, evolutionary relationships, *acting elements* and various abiotic stresses. Studies have shown that the *GhLCYε-3* gene affects the drought resistance of cotton by regulating the content of lutein MDA and PRO content. The results of this study further elucidate the relationship between LCYs and lutein and enhanced stress resistance.

In order to explore the relationship between lutein with germination, fresh and root length, this experiment was designed. Lutein content analysis indicated a positive correlation between germination potential and fresh weight with lutein, and a negative correlation with root length. Lycopene cyclases (LCYs), crucial enzymes regulating lutein production, have been shown to increase lutein accumulation upon overexpression [39]. Based on previous research, we analyzed LCYs in cotton to explore the relationship between LCYs regulating lutein. The research shows that the length of this gene protein is 487–769 kD, which is consistent with previous studies [40]. Subcellular prediction showed localization on chloroplasts, possibly lycopene cyclase controlling lutein production, while lutein and chlorophyll belong to the photosynthetic system, and analysis of LCYs *cis-acting* elements found that the LCYε group had more *cis-acting* elements [41]. The lycopene cyclase family is divided into two branches, LCY-ε and LCY-β, which is consistent with previous studies, and interestingly, in algae plants, LCYs is divided into three groups, CrtL-b, CrtL-e, and CrtL, speculating that CrtL may have other important roles [42] LCYs genes were found to be closely related to four types of cotton, and it may be that these four cotton species evolved from a common ancestor and had more homologous genes [43]. For instance, *GhLCYε-3* and *GhLCYε-1* are a pair of homologous genes. We found that the four cotton species of LCYs protein were closely related to cocoa, but were distantly related to maize. While the LCYs protein of the four cotton species closely relates to cocoa, it is distantly related to maize, possibly due to their common ancestry in the mallow family. LCYs was found to be more conserved during evolution, and it is speculated that such a highly conserved LCYs is important for maintaining the normal function of organisms, so the gene is not easily mutated and is highly preserved [44].

Chromosome mapping analysis further supports the evolutionary link of LCYs,with genes evenly distributed on four chromosomes. The similarity in gene positions strengthens the notion that cotton's heterotetraploid state resulted from doubling chromosomes after A-genome diploid hybridization [45]. LCY-ε and LCY-β groups, despite a large number of similar motifs, showcase an interesting case where (GhLCYε-2, GbLCYε-2) possesses 3–4 motifs less, hinting at potential lost functions during evolution. Notably, the LCYε group containing introns contrasts with the intron absence in the LCYβ group, indicating potential rapid development through reverse transcription or replication [46]. Introns are the parts that are cut off during messenger RNA processing and are not present in mature messenger RNA. Non-coding regions in GrLCYε-1, GrLCYβ-2, GaLCYβ-1, and GrLCYβ-2 reveal potential locations for LCY transcription factors [47]. Since promoters are typically located in non-coding regions, non-coding regions have regulatory functions for gene expression. It has been observed in non-coding regions of *GrLCYε-1, GrLCYβ-2, GaLCYβ-1, and GrLCYβ-2*, and we speculate that transcription factors of LCY may be located in these regions [48]. Protein prediction showed that the GhLCYε-3 pit regulated the content of lutein and ABA by interacting with 10 proteins, thereby completing the response to drought stress. Quantitative fluorescence analysis showed that the expression level of LCYs was observed to be quite different from that of the control after PEG treatment of cotton, and it was speculated that LCYs gene played an important role in responding to drought stress in cotton. Two enzymes, lycopene β cyclase and lycopene ε cyclase, are found in most species and play an important role in regulating the abundance of carotenoids [12], [49]. It is hypothesized that lycopene cyclase ε in cotton can also regulate the content of carotenoids, thereby improving the stress resistance of plants [42].

**Fig. 11:**
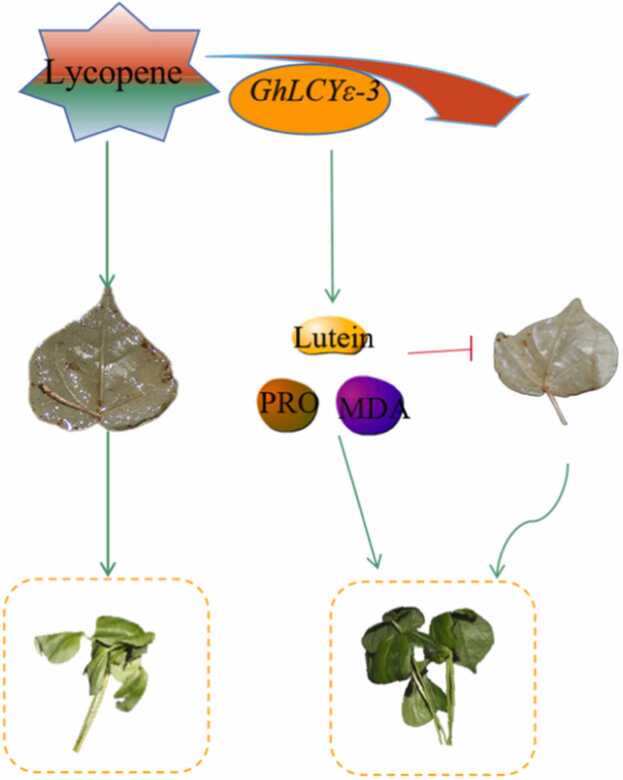
Mechanism of GhLCYε-3 regulating cotton response to drought stress. Annotation：lycopene (Synthetic substrates).GhLCYε-3 (Lycopene ε-cyclase) lutein (Clear ROS) MDA (Malondialdehyde) PRO (Proline).

By comparing LCYs sequences in *Arabidopsis*, we identified a putative *GhLCYε-3* gene for VIGS validation. Lutein serves as an effective antioxidant to protect cells from oxidative damage caused by ROS [50]. Our research establishes a molecular mechanistic model was developed, unveiling *GhLCY-3*'s role in controlling the synthesis of ABA and lutein (Fig. 11). Drought stress induces an increase in ROS, leading to observed lutein cycle disorders in silent plants, resulting in ROS accumulation and wilted leaves[51]. The inverse relationship between lutein and MDA levels, coupled with elevated MDA and Pro levels in VIGS plants, indicates increased stress and severe damage to cell membranes[52]. The expression of lutein and ABA-related genes may be impacted by the interaction between lycopene β*-*cyclase and lycopene ε*-*cyclase [53]. *GhLCYε-3* is a key gene in lutein synthesis. exhibits up-regulated expression under drought stress, increasing lutein content and enhancing drought resistance by reducing oxidative damage (Fig. 11). The signaling mechanism during plant growth and development is activated by adversity stress, and this adaptation mechanism of plants to adversity leads to a variety of molecular and physiological changes. In conclusion, the regulation of lutein, MDA and PRO by *GhLCYε-3* is an important response mechanism of cotton to drought stress, which provides valuable insights for improving cotton stress resistance.

In this research, from lutein and stress resistance, material screening, bioinformatics analysis of LCYs family, gene mining and functional verification, it was observed that the *GhLCYε-3* gene responded to cotton drought stress, and its function was verified by VIGS. However, this experiment also has certain limitations, the research is limited to one material and one treatment. Further investigations are warranted to explore the interaction and regulation of *GhLCYε-3* with other genes involved in stress response pathways under different abiotic stresses.

## 5 Conclusion

In this research, aimed at unraveling the regulatory role of lutein in enhancing cotton's drought resistance, conducted a comprehensive evaluation of LCYs gene structure, phylogeny, and expression patterns. Fluorescence quantitative experiments showed that GhLCYs genes responded to various abiotic stresses, and the function of *GhLCYε-3* was preliminarily verified by VIGS. In sum, this research provides new insights into the molecular mechanism of gene function and regulation of lycopene cyclase in cotton. The findings not only aid in enhancing the resistance and quality of cotton under drought stress but also offer valuable guidance for cotton cultivation in arid regions.

## Ethics approval and consent to participate

Not applicable.

## Consent to publication

All authors consent this version for publication.

## Funding

This work was supported by The 10.13039/501100012166National Key Research and Development Program of China (2023YFD1200300).

## CRediT authorship contribution statement

YW conceived the experiments. NK, LX, LS, LF, ZY, and HY performed the experiments. NK, ZY, FY, CR and HH analyzed the data. NK wrote the initial draft of the manuscript. CX, WJ, WS, GL, ZL and WY supervised the project. All the authors read and revised the final manuscript.

## Acknowledgements

The authors acknowledge technical assistance of Cotton Research Institute of the Chinese Academy of Agricultural Sciences.

## Declaration of Competing Interest

The authors declare that the research was conducted in the absence of any commercial or financial relationships that could be construed as a potential conflict of interest.

## Data Availability

The datasets generated and/or analyzed during the current study are available in the CottonFGD (https://cottonfgd.org/). Additional: Supplementary Table S1.
